# Hyperactivation of Oncogenic JAK3 Mutants Depend on ATP Binding to the Pseudokinase Domain

**DOI:** 10.3389/fonc.2018.00560

**Published:** 2018-12-03

**Authors:** Juuli Raivola, Henrik M. Hammarén, Anniina T. Virtanen, Vilasha Bulleeraz, Alister C. Ward, Olli Silvennoinen

**Affiliations:** ^1^Faculty of Medicine and Life Sciences, University of Tampere, Tampere, Finland; ^2^School of Medicine, Deakin University, Geelong, VIC, Australia; ^3^Centre for Molecular and Medical Research, Deakin University, Geelong, VIC, Australia; ^4^Fimlab Laboratories, Pirkanmaa Hospital District, Tampere, Finland; ^5^Institute of Biotechnology, University of Helsinki, Helsinki, Finland

**Keywords:** JAK kinase, nucleotide binding, pseudokinase, cytokine, leukemia

## Abstract

Janus kinase 3 (JAK3) tyrosine kinase has a central role in the control of lymphopoiesis, and mutations in JAK3 can lead to either severe combined immunodeficiency or leukemia and lymphomas. JAK3 associates with the common gamma chain (γc) receptor and functions in a heteromeric signaling pair with JAK1. In IL-2 signaling JAK1 is the effector kinase for STAT5 phosphorylation but the precise molecular regulatory mechanisms of JAK1 and JAK3 and their individual domains are not known. The pseudokinase domain (JAK homology 2, JH2) of JAK3 is of particular interest as approximately half of clinical JAK3 mutations cluster into it.

In this study, we investigated the role of JH2s of JAK1 and JAK3 in IL-2R signaling and show that STAT5 activation requires both JH1 and JH2 of JAK1, while both JH1 and JH2 in JAK3 are specifically required for the cytokine-induction of cellular signaling. Characterization of recombinant JAK3 JH2 in thermal shift assay shows an unstable protein domain, which is strongly stabilized by ATP binding. Unexpectedly, nucleotide binding to JAK3 JH2 was found to be cation-independent. JAK3 JH2 showed higher nucleotide binding affinity in MANT-ATP and fluorescent polarization competition assays compared to the other JAK JH2s. Analysis of the functional role of ATP binding in JAK3 JH2 in cells and in zebrafish showed that disruption of ATP binding suppresses ligand-independent activation of clinical JAK3 gain-of-function mutations residing in either JH2 or JH1 but does not inhibit constitutive activation of oncogenic JAK1. ATP-binding site mutations in JAK3 JH2 do not, however, abrogate normal IL-2 signaling making them distinct from JH2 deletion or kinase-deficient JAK3. These findings underline the importance of JAK3 JH2 for cellular signaling in both ligand-dependent and in gain-of-function mutation-induced activation. Furthermore, they identify the JH2 ATP-binding site as a key regulatory region for oncogenic JAK3 signaling, and thus a potential target for therapeutic modulation.

## Introduction

The family of Janus kinases (JAK1, JAK2, JAK3, and TYK2) are key mediators of cytokine signaling that regulate hematopoietic cell development, cellular metabolism and immune responses. Within the JAK family, JAK3 is unique in its restricted expression to hematopoietic lineages and specific interaction only with the common γ chain (γc) cytokine receptor (1). In cytokine signaling, JAK3 functions in a heterodimeric pair with JAK1, which binds to the cytokine specific receptor β chains. The JAK1-JAK3 pair transmit signals emanating from interleukin (IL)−2, −4, −7, −9, −15, and −21 that regulate development and activation of lymphoid lineage cells (2). The critical role of JAK3 in lymphoid cells is highlighted by the phenotypes of JAK3 activating mutations causing different types of leukemia and lymphomas (3–5), as well as by JAK3 deficiencies that cause severe combined immunodeficiency (SCID); a condition resulting from profound defects in mature T cells and B cells and innate lymphoid cells including NK cells (6–8).

JAKs share a conserved domain structure consisting of an N-terminal FERM domain and a Src homology 2 (SH2)-like domain that comprise the receptor-binding unit, followed by a pseudokinase domain (JAK homology 2, JH2) and a tyrosine kinase domain (JH1) (1, 9, 10). Signaling is initiated by cytokine binding to the extracellular domain of their respective receptors that induces receptor dimerization/oligomerization or a conformational shift juxtaposing the JAKs and allowing their activation through trans-autophosphorylation of the activation loop tyrosine residues. Following activation, JAKs phosphorylate tyrosine residues in the receptor chains leading to recruitment and subsequent phosphorylation of downstream effectors such as the signal transducers and activators of transcription (STATs) (1, 2, 10). In IL-2 receptor (IL-2R) signaling, JAK1 has been shown to play a dominant role in early cytoplasmic activation events where JAK1 is primarily driving STAT5 phosphorylation (11), while JAK3 is required for sustained activation during T-cell proliferation (11, 12). Both functions are dependent on the catalytic activity of the JH1 domains in JAK1 and JAK3, respectively. However, several aspects of IL-2R signal regulation are still elusive, especially with respect to the roles of the pseudokinase domains.

JAK kinases are constitutively associated with cytokine receptors and the activity of the JAK–STAT pathway needs to be tightly regulated at multiple levels to suppress signaling in the absence of cytokine stimulation and to allow rapid, transient activation upon stimulation (9, 10). Regulation relies primarily on control of the JH1 tyrosine kinase activity through intra- and intermolecular mechanisms (1, 9), and for the former, the function of JH2 is important, although still somewhat enigmatic. Genetic studies provide compelling evidence for the regulatory function of JH2 as disease-causing mutations strongly cluster in JAK JH2s (13, 14). The best characterized example is JAK2 where somatic mutations in JH2 (JAK2 V617F and >30 other mutations) result in constitutively active JAK2 and are responsible for approximately 80% of myeloproliferative neoplasms (MPN) and less frequently for different types of leukaemias including acute lymphoblastic leukemia (ALL), acute megakaryoblastic leukemia (AMKL), and acute myeloid leukemia (AML) (5, 14, 15). In JAK3, approximately half of all clinical mutations localize to JH2 (13, 16).

JAK3 JH2 has two interesting and unique features compared to the other JAKs: it is the only JH2 harboring both activating and inactivating clinical mutations, and secondly it is the only JH2 where the prototypic JH2 gain-of-function mutation (JAK2 V617F, JAK3 homolog M592F) does not cause hyperactivation (17). Some inhibitory SCID mutations result in truncated proteins, but several mutations cause amino acid changes in either the C- and N-lobes of JH2 (e.g., E481G, del482-596, R582W, G589S, C759R). More recently, several activating JAK3 JH2 mutations (e.g., M511I, A572V, R657Q, V722I) have been found in acute myeloid and lymphoblastic leukemia (AMKL, T-ALL, AML, natural killer cell lymphoma, acute megakaryoblastic leukemia, T-cell prolymphocytic leukemia, juvenile myelomonocytic leukemia and natural killer T cell lymphoma) (3, 4, 18). Although genetic information from patients provide compelling evidence for the importance of JH2 regulatory function, they do not provide molecular explanations of the underlying mechanisms of normal or pathogenic JH2 function.

JAK JH2s show significant sequence homology with classical protein kinases but several conserved functional residues or motifs are missing or altered. Most importantly, the HRD motif, including the catalytic base aspartate (D) is replaced by HGN resulting in a presumably catalytically inactive kinase (hence “pseudokinase”) (19–21). Structural and biochemical analysis of JAK1, JAK2 and TYK2 JH2s have shown that despite the sequence variations, the JH2 domains adopt a typical two lobed kinase-fold able to bind ATP (22–24). JAK2 and TYK2 JH2 structures have been solved with ATP and they show a non-canonical binding mode with ATP complexed to only a single cation instead of the usual two (23, 25). In addition, the canonical ionic interaction between the β3 lysine (K556 in JAK3 JH2; K72 in the archetypal cAMP-dependent protein kinase PKA) and αC (E91 in PKA, alanine in JAK JH2s) is replaced by a β3 lysine–aspartate interaction to the DFG motif (DPG in JAK JH2s) (19, 23, 25–27).

Early biochemical studies suggested a dual regulatory or “switch” function for JH2 where the domain is required for maintaining JAKs inactive in the absence cytokine stimulation, as well as mediating cytokine-induced signaling (27–31). Recent structural information from JAK2 (structural model) and TYK2 (crystal structure) provide molecular basis for the JH2 inhibitory function and show that JH2 interacts with the hinge-region of JH1 and stabilizes the JH1 in an inactive conformation (27, 32). Most gain-of-function JAK2 disease mutations localize in this interface and are predicted to disrupt the JH1–JH2 autoinhibitory interaction resulting in constitutive activation (27, 28, 33). The nature of the activating function of JH2 in cytokine signaling and in oncogenic JAK3 mutations is currently unknown.

## Materials and Methods

### Protein Expression and Purification

Expression: JAK3 JH2 (residues 511–790) was cloned into the pFASTBAC1 vector (Invitrogen) with a C-terminal His_6_ tag and expressed as a fusion protein in *Spodoptera frugiperda* (*Sf* 9) cells. For protein expression, cells were infected with 10% (v/v) virus supernatant, grown for 48 h and collected by centrifugation.

Ni-NTA purification: Cell pellets containing JH2-His fusion protein were resuspended in lysis buffer containing 20 mM Tris-HCl (pH 8.0), 500 mM NaCl, 20% (v/v) glycerol, 0.5 mM TCEP and 20 mM imidazole, supplemented with protease inhibitors cocktail, lysed using a cell disruptor (Sonics) and clarified by centrifugation for 1 h at 10,000 g. The supernatant was incubated for 2 h with prewashed Ni-NTA beads (Qiagen) with gentle rotation at 4 °C. The beads were extensively washed with a buffer supplemented with 40 mM imidazole, and the fusion protein was eluted with 150 mM imidazole buffer. Fractions containing the fusion protein were pooled and buffer-exchanged by several dilution-concentration cycles using 10 K Amicon Ultra centrifugal filters.

Size exclusion chromatography: Ni-NTA purified, buffer-exchanged and concentrated proteins were loaded into Superdex 75 10/300 GL column (GE Healthcare Life Sciences) equilibrated with 20 mM Tris-HCl (pH 8.0), 500 mM NaCl, 20% (v/v) glycerol and 0.5 mM TCEP. The fractions including the protein were collected and concentrated for further analysis.

### Differential Scanning Fluorimetry

Protein concentration of 3 μM was used for the measurement of the melting temperature (Tm). Reactions were done in the buffer used in the SEC. Addition of other components (ATP, salts) were taken into account by including 2X buffer, if needed. Signal arising from the fluorescent dye Life Technologies SYPRO^TM^ Orange Protein Gel Stain, 4X working solution from 5000X concentrate in DMSO) was measured with a conventional Real-Time PCR system (Bio-Rad CFX), 1°C per min from 4 to 96°C. Results were transferred and normalized in Microsoft Excel and the Tm was derived from the sigmoidal function using GraphPad Prism version 5.02 for Windows, GraphPad Software, La Jolla California USA, https://www.graphpad.com/.

### MANT-ATP Binding Assay

The steady-state fluorescence of MANT-labeled ATP was measured as described in Mysore et al. (34). Briefly, the fluorescently labeled MANT (2′/3′-(N-methyl-anthraniloyl)-ATP was titrated against 1.5 μM recombinant JAK3 JH2. In addition, the protein was titrated as 0, 0.05, 0.25, 1.25, and 6.26 μM against 0.25 μM MANT-ATP. FRET signal was measured with QuantaMaster PTI Fluorometer and the data was analyzed using GraphPad Prism version 5.02 and Microsoft Excel. Protein titrations were done in duplicate and in MANT-ATP titration in triplicate.

### Fluorescence Polarization Assay

Fluorescence polarization measurements were performed in black 384-well plates (ProxiPlate-384 F Plus, PerkinElmer) at sample volume of 5 μl/well on PerkinElmer Envision plate reader using 480 nm excitation and 535 nm emission filters. Fluorescent tracer, Bodipy FL labeled JNJ-7706621 [compound 5 in (35)] was used at 1.5 nM concentration. Recombinant JAK1 JH2 553-836-His, JAK2 JH2 503-827-His, JAK3 JH2 511-790-His, and TYK2 JH2 564-876-His proteins were used at concentrations (2 nM JAK1, 150 nM JAK2, 1 μM JAK3, 20 nM TYK2) dependent on protein-tracer dissociation constants. The assays were performed in a buffer consisting of 20 mM Tris-HCl pH 8.0, 150 mM NaCl, 20% glycerol, 0.01% Brij-35, and 2 mM DTT. ATP was titrated at concentration range of 5 nM−5 μM and fluorescence polarization values obtained were fitted against log[ATP] in GraphPad Prism to yield IC50 values. Assays were performed in triplicate.

### Plasmids and Mutagenesis

C-terminally HA-tagged full length JAK1, JAK3, and STAT5 cloned into pCIneo mammalian expression plasmid were used for transfections. Mutations into JAK1 and JAK3 were introduced by site-directed mutagenesis using QuikChange-protocol with specifically designed primers. The method was also used for the domain deletion constructs.

### Cell Culture, Transfection, and Immunoblotting

JAK1 and JAK3 deficient U4C fibrosarcoma cells stably expressing IL-2Rγ and -β receptors were kindly provided by Dr. Claude Haan, (Life Sciences Research Unit, Signal Transduction Laboratory, University of Luxembourg, Luxembourg, L-1511, Luxembourg) and Hans-Günter Zerwes (Novartis Institute for Biomedical Research). Cells were cultured in Dulbecco's Modified Eagle's Medium (DMEM) supplemented with 10% fetal bovine serum, 1% pen strep and 1% glutamine and antibiotics: puromycin, zeozin and blasticidin for maintaining the IL-2Rγ and -β expression.

Transient transfections were done with full-length or mutant human JAK1-HA (75 ng), human JAK3-HA (75 ng) and human STAT5A-HA (1.5 ng) using FuGENE HD (Promega) according to the manufacturers' instructions. After 34 h cells were starved in serum-free medium overnight and stimulated for 15 min with 100 ng/ml of human IL-2. After stimulation, cells were lysed into Triton-X cell lysis buffer and centrifuged, and the supernatant used for SDS/PAGE and immunoblotting. Blots were double-stained with phosphospecific antibodies (P-Stat5 (Y694), Cell Signaling Technology and anti-phospho-JAK1 (Tyr1022/Tyr1023), Millipore) and anti-HA (Aviva Systems Biology OAEA00009) and detected with a mix of IRDye-labeled secondaries. The STAT5 (HA) signals were used for normalization of phospho-STAT5 levels. Blots were quantified using a LI-COR Odyssey CLx imaging system. A minimum of three independent experiments were performed for each condition.

### JAK3 Homology Modeling

A JAK3 homology model was generated using the SWISS-MODEL server with the TYK2 structure (Protein Data Bank identification 4OLI) as the modeling template. Graphical presentations were generated using the PyMOL Molecular Graphics System (DeLano Scientific, San Carlos, CA).

### Luciferase and Dual Luciferase Assays

STAT5 transcriptional activity was assessed by measuring the luciferase expression (SPI Luc 2) driven by a STAT5 responsive promoter from growth hormone regulated serine protease inhibitor, as described previously (27). U4Cγβ cells were transfected with indicated DNA constructs including SPI-Luc2 and β-galactosidase reporter plasmids. The latter was co-transfected as an internal transfection control. Transient transfections were done in 96 well plates with FuGENE HD (Promega) according to manufactures instructions. 42 h after transfection, cells were stimulated (in starvation media) or starved for 5 h after which luciferase assays were analyzed using the dual luciferase reporter assay system (Promega) according to manufactures instructions. Luciferase values were measured with EnVision 2104 Multilabel Reader (Perkin Elmer). The results are presented as relative luciferase activity (RLU) corresponding to the firefly luciferase light emission values divided by renilla luciferase light emission values.

In addition to Renilla luciferase (pRLTk; Promega), the β-galactosidase reporter was also used, and similar results were obtained with both reporters. Briefly, cells were washed twice with PBS and 20 μl of 1X Luciferase lysis buffer was added to the wells. 10 μl of the lysate was used for luminescence measurement with 75 μl luciferase reagent. β-galactosidase signal was measured by adding 50 μl of ONPG per well and the absorbance signal values were used for normalization of the luciferase luminescence values.

### *In vivo* Studies in Zebrafish

Embryos derived from *jak3*^+/−^ in-crosses were injected with 100 pg/nl *in vitro* transcribed capped mRNA encoding zebrafish JAK3 wild-type (WT), and M511I, I535F and M511+I535F mutants. At 5 dpf they were fixed and subjected to whole-mount *in situ* hybridization (WISH) with anti-sense *rag1* probe and imaged and quantified as described (36), with only *jak3*^−/−^ embryos, as determined by post-WISH genotyping, used in the analysis. In other experiments, embryos from *lck*::GFP zebrafish were injected and rag1 expression analyzed using RT-PCR.

## Results

### Both JH1 and JH2 in JAK1 and JAK3 are Required for Functional IL-2–STAT5 Signaling

In order to investigate the functional role of JAK3 JH2 in IL-2R signaling, we used the human fibrosarcoma U4Cγβ cell line that stably expresses IL-2Rγ and IL-2Rβ but lacks expression of JAK1 and JAK3 (11). A functional IL-2R signaling cascade was reconstituted by ectopic expression of JAK1 and JAK3, which allowed analysis of JAK1/JAK3 mutants. Optimization of experimental conditions showed that expression of JAK1 alone induced strong basal activation of STAT5-driven luciferase reporter SPI-Luc2 (27), which was, however, unresponsive to IL-2 stimulation (Supplementary Figure 1A). Expression of JAK3 alone showed no basal STAT5 transcriptional activity and no responsiveness to IL-2. IL-2-induced STAT5 activation was achieved only by expression of both JAK1 and JAK3, which is in line with previous studies (3, 11, 37).

Immunoblot analysis of STAT5 Y694 phosphorylation (pSTAT5) from U4Cγβ cells transfected with JAK1 and JAK3 concurred with STAT5 transcriptional activity showing IL-2-independent phosphorylation by JAK1 alone, lack of pSTAT5 by JAK3 alone and requirement for both JAK1 and JAK3 for IL-2-responsiveness (Supplementary Figure 1B). Collectively these results imply JAK1 as the immediate downstream effector kinase and JAK3 as a regulator/activator in the IL-2R signaling complex, thus confirming previous reports (3, 11, 37). However, the underlying molecular mechanisms and regulatory domains in JAK1–JAK3 heterodimer signaling are still largely unknown and, in this context, we wanted to analyze specifically the roles of JH2s.

**Figure 1:**
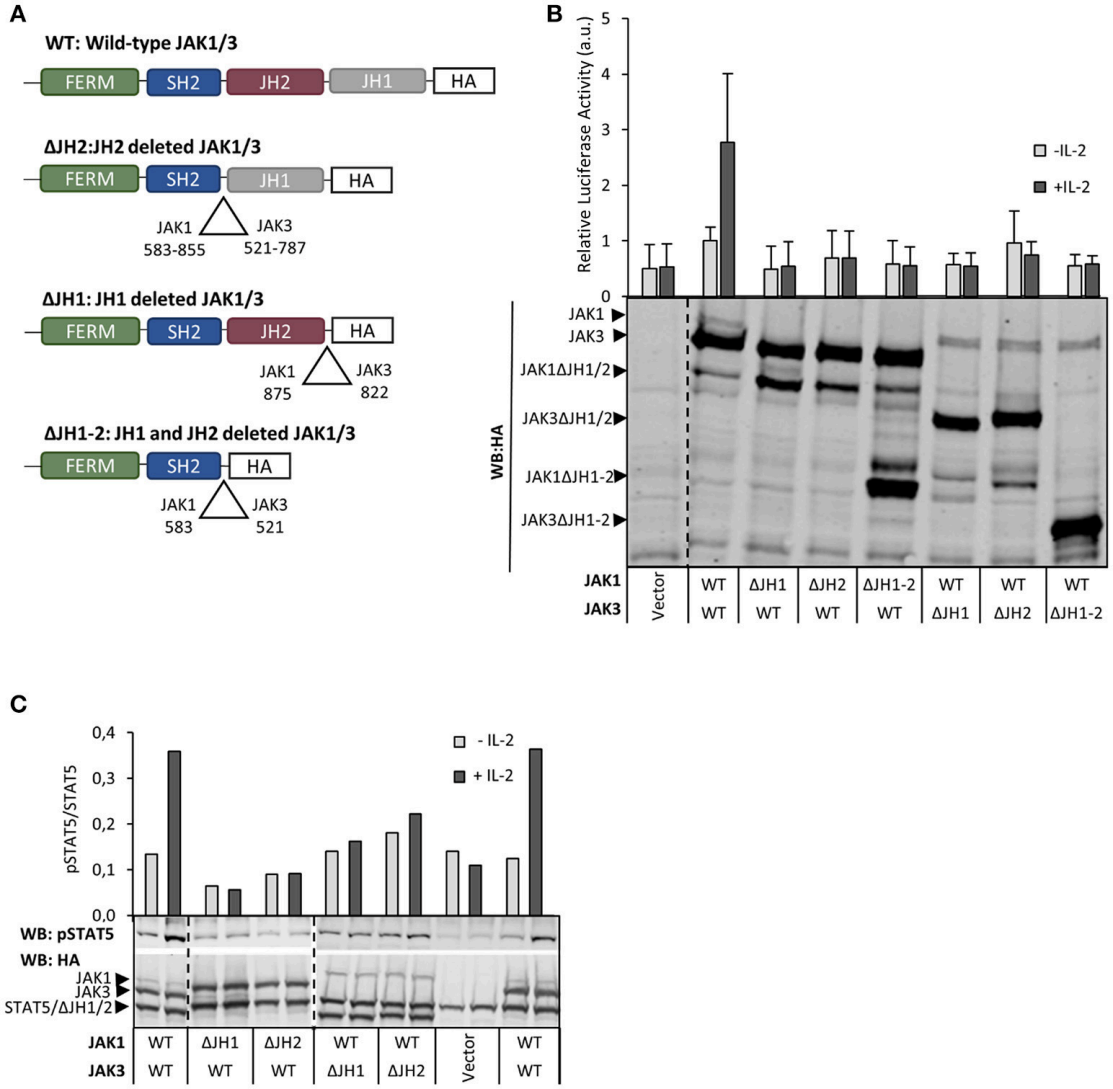
Both JH1 and JH2 in JAK1 and JAK3 are required for functional IL-2 signaling. **(A)** Schematic presentation of JAK1 and JAK3 wild-type (WT) and domain deletion constructs. **(B)** Both JH1 and JH2 of JAK1 and JAK3 are required for IL-2 dependent STAT5 activation. U4Cγβ cells were transfected with JAK1 WT, JAK3 WT and domain deletion constructs together with STAT5 responsive reporter SPI-luc2 (28), either with Renilla or pRL-TK control vector (see Materials and Methods) and starved/stimulated. Similar results were obtained with both control vectors. Data shows averages and SD from normalized values combined from three independent experiments, each having triplicate samples. The expression of JAK1/JAK3 constructs was analyzed by anti-HA Western blotting. **(C)** IL-2-induced pSTAT5 requires both JH1 and JH2 in JAK1 and JAK3. STAT5 phosphorylation and expression of JAK1, JAK3 and STAT5 were detected from cell lysates from transfected U4Cγβ cells by immunoblotting with HA- and pTyr694 STAT5 antibodies. Similar results were obtained in three independent experiments.

In order to investigate the roles of different JH domains we produced deletion constructs for JH1 and JH2 domains in JAK1 and JAK3 (Figure 1) In accordance with the critical role of JAK1 kinase activity in IL-2R–STAT5 signaling (see above), deletion of JH1 in JAK1 (JAK1 ΔJH1) abolished both basal and IL-2-induced pSTAT5 and STAT5 reporter activity. Similarly, deletion of JH1 in JAK3 abolished IL-2-inducibility but retained basal activity mediated by JAK1. Interestingly, deletion of JH2 in JAK1 also completely abrogated IL-2R signaling activity, and in JAK3 deletion of JH2 or both JH1 and JH2 prevented IL-2-inducibility without affecting basal activity (Figures 1B,C). These results indicate that, in addition to the kinase domains, the pseudokinase domains in JAK1 and JAK3 are also required for IL-2R signaling.

**Figure 2:**
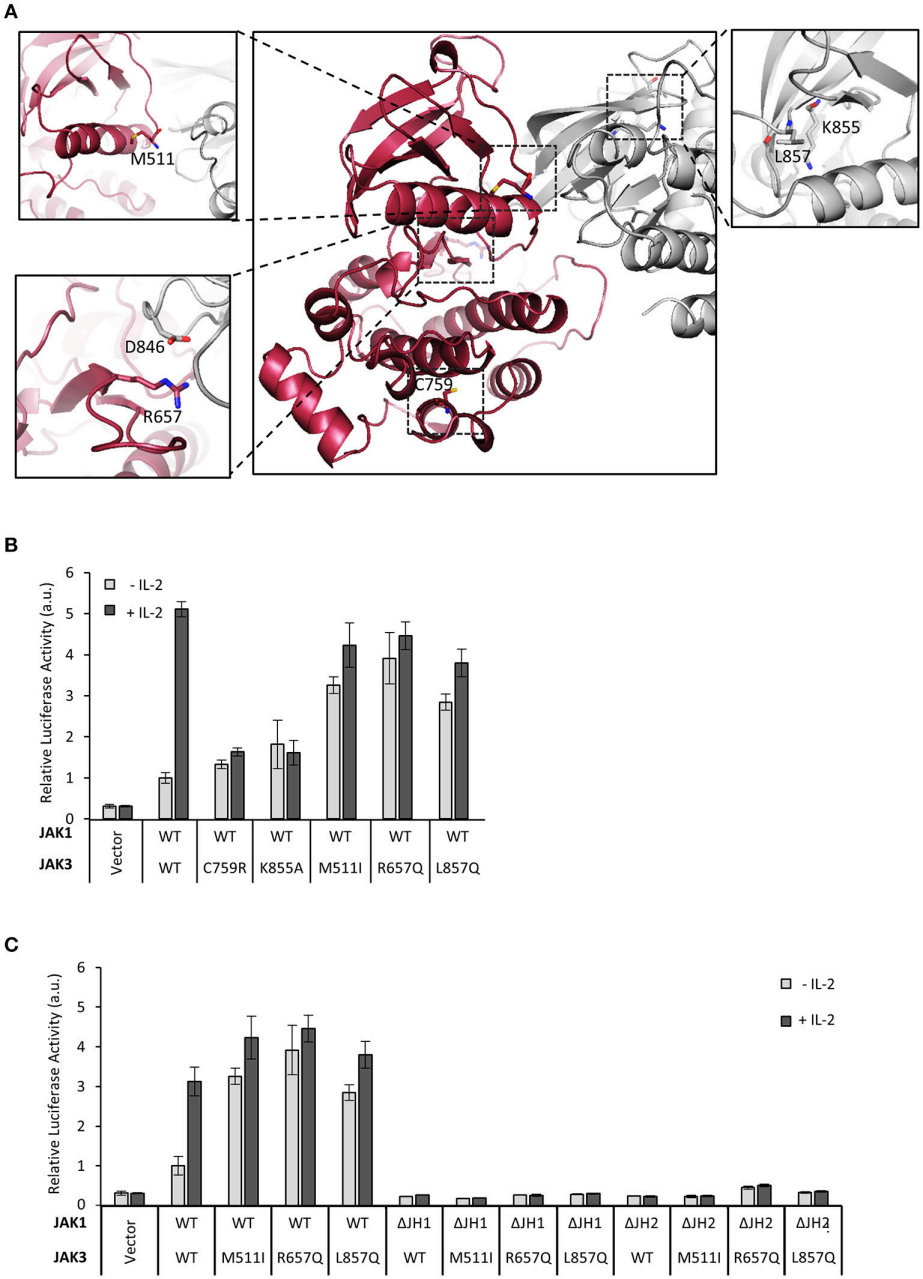
Mutations in JAK3 JH2 can alter the function of the IL-2 pathway. **(A)** Localizations of the JAK3 mutants used in this article. Homology model of JAK3 JH2-JH1 (based on the TYK2 structure in Protein Data Bank (PDB) code: 4OLI) and the JAK3 JH1 structure [PDB code: 1YVJ (38)] were aligned with the JH1-JH2 structure of TYK2 (PDB code: 4OLI). JH2 is shown in red and JH1 in gray. Close-ups of the mutations are shown in subpanels. C759 is shown in the main picture. In the lower left panel, the (possible) interaction pair R657–D864 (in JH1) is shown. In the right panel, ATP was aligned from ATP-bound form of PKA [PDB code: 1ATP (39)]. **(B)** JAK3 JH2 C759R mutation efficiently inhibits IL-2 signaling and activating mutations in JAK3 JH2 and JH1. Luciferase assay of STAT5 activation in U4Cγβ cells transfected with JAK1 WT and JAK3 point mutations, SPI-luc2 reporter and β-galactosidase control. Values were normalized to JAK1 WT and JAK3 WT transfected sample Errors bars show SD of triplicate samples. Similar results were obtained using pRL-TK as a control. **(C)** Activating JAK3 mutants rely on both JH1 and JH2 of JAK1 for constitutive downstream signaling. SPI-luc2 reporter was used to detect the activation of STAT5 in U4Cγβ cells. Cells were transfected with WT or domain deletion JAK1 and WT or constitutively active JAK3 mutants. β-galactosidase plasmid was used as an internal control for the transfection efficiency. Values were normalized to WT JAK1 and JAK3. Error bars are SD of triplicate samples.

Next, we extended our studies on the regulatory function of JH2 to clinical JAK3 mutations. As the structure of JAK3 JH2 has not been solved, we produced a homology model based on the TYK2 JH2-JH1 structure (Protein Data Bank (PDB) code: 4OLI, see Figure 2A) (27). We analyzed pathogenic inactivating mutant C759R found in SCID patients as well as activating JAK3 mutants M511I and R657Q in JH2 and L857Q in JH1 (list of mutations; see Table 1). The activating mutants are found in T-ALL patients and reported to be constitutively active in cellular assays and result in leukemic phenotypes in mouse models (3). Mutations were studied in the IL-2–STAT5 pathway in U4Cγβ cells co-expressed with wild-type JAK1. The JAK3 C759R inhibited basal and IL-2-induced STAT5 activation as efficiently as the kinase-inactive JAK3 JH1 mutation K855A (Figure 2B). JAK3 M511I and R657Q showed similar, cytokine-independent STAT5 activation as the JH1 L857Q mutant (Figures 2B,C, Supplementary Figure 2C).

**Table 1:** Mutations used in this study.

**JAK**	**Mutation**	**Domain**	**Effect**	**References**
JAK3	M511I	JH2	Found in T-ALL patients, constitutively active	(3)
	R657Q	JH2	Found in T-ALL patients, constitutively active	(3)
	L857Q	JH1	Found in T-ALL patients, constitutively active	(3)
	C759R	JH2	Found in SCID patients, inhibits JAK3 kinase activity and reduces STAT5 activity	(31)
	K855A	JH1	Catalytic lysine, kinase dead
	K566A	JH2	Homologous to K855A, disrupts ATP binding to JH2
	I535F	JH2	Homologous to JAK2 I559F, disrupts ATP binding to JH2	(24)
JAK1	V658F	JH2	Homologous to JAK2 V617F, constitutively active	(17)
	I535F	JH2	Homologous to JAK2 I559F, disrupts ATP binding to JH2	(24)

Most activating JAK3 mutants have been shown to depend on JAK1 for signaling (3), and we sought to define the JAK1 domains required for constitutive IL-2 signaling. Activating JAK3 mutants were transfected with either wild-type JAK1, JAK1 ΔJH2 or JAK1 ΔJH1 into U4Cγβ cells. All tested JAK3 mutants residing in either JH1 or JH2 were found to depend on normal JAK1 activity and showed increased basal STAT5 activation only in the presence of full-length JAK1 (Figure 2B, Supplementary Figure 2A); deletion of either JH1 or JH2 of JAK1 completely abrogated signaling as measured by pSTAT5 and STAT5 luciferase assays (Figure 2C, Supplementary Figures 2B,C). Taken together, these data show that the analyzed oncogenic JAK3 JH2 and JH1 mutations require both JH1 and JH2 domains of JAK1 for constitutive signaling.

### Characterization of Recombinant JAK3 JH2 Shows a Domain Capable of Tight, Cation-Independent Nucleotide Binding

**Figure 3:**
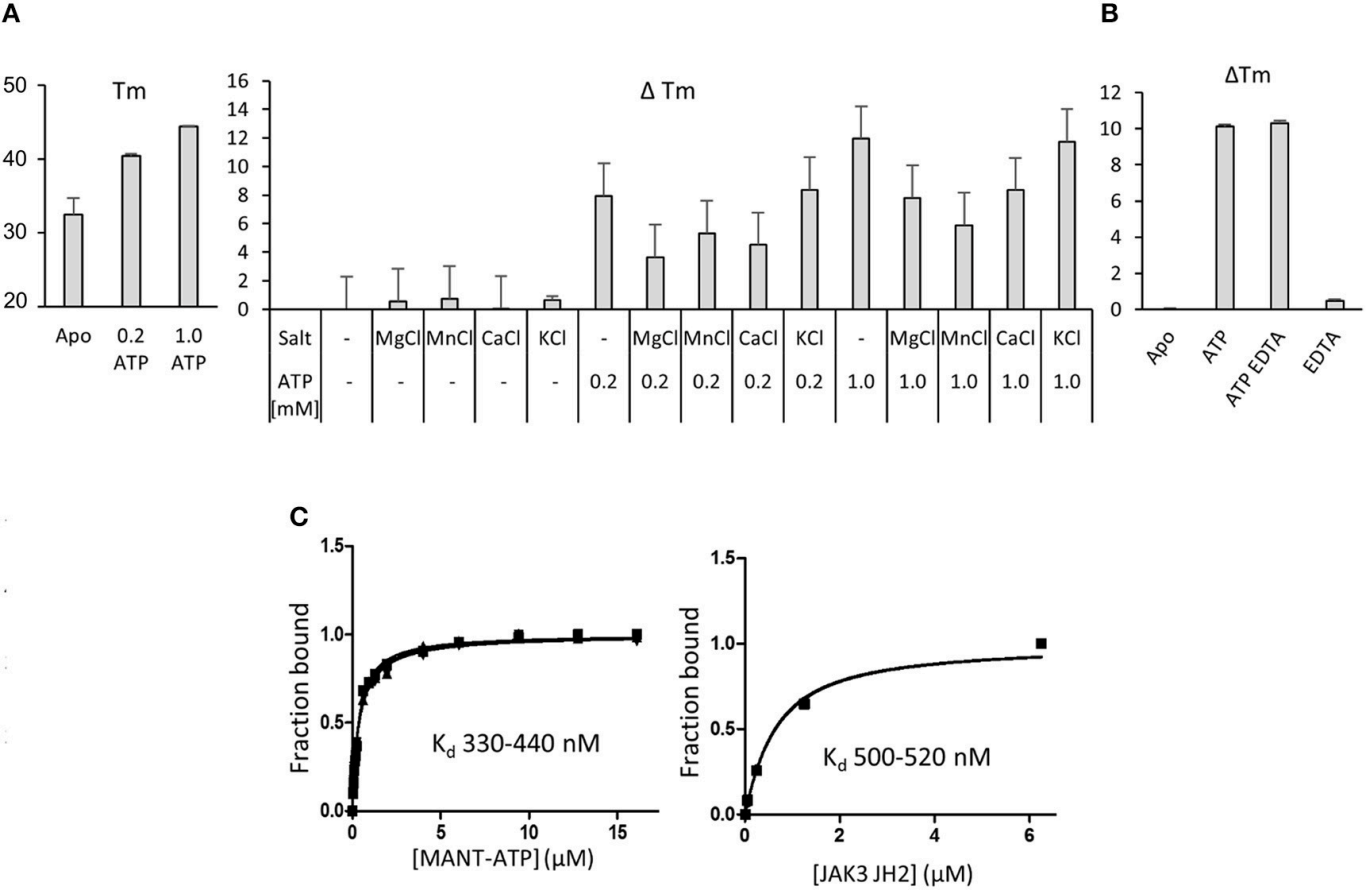
Nucleotide binding to recombinant JAK3 JH2. **(A)** JAK3 JH2 binds ATP without cations. The effect of ATP on Tm of JAK3 JH2 is shown in the left panel. The following graph shows changes in the melting temperature (ΔTm) in JAK3 JH2 in the presence of different cations and ATP. **(B)** Chelating agent does not affect ATP-induced increase on Tm of JAK3 JH2. DSF analysis performed in the presence of ATP (1 mM) and chelating agent EDTA (2 mM). **(C)** MANT-ATP binding assay with recombinant JAK3 JH2. Both protein and MANT-ATP were titrated and both experiments gave similar Kd in the sub-micromolar range. Left panel: titrating MANT-ATP to 1.5 μM JAK3-JH2. Kd range 330–440 nM. Right panel: titrating JAK3 JH2 to 0.25 μM MANT-ATP, Kd 500 nM. Protein concentrations were 0; 0.05; 0.25; 1.25 and 6.25 μM. In both figures, the Y-axis is the fraction of ligand-bound protein in relation to the total protein amount ([PL/Ptot]). The MANT-ATP titration was repeated three times and all experiments are plotted in the lower left panel. Protein titration was repeated twice and both experiments are blotted in the figure.

Pseudokinases are generally considered to be inactive kinases but an estimated 30–40% of pseudokinases, including JAK1, JAK2 and TYK2 JH2, bind nucleotides (21). The function of JAK3 JH2 has not been analyzed and for biochemical characterization recombinant JAK3 JH2 (residues 511–790 with C-terminal His_6_-tag) was expressed in insect cells and purified with affinity chromatography followed by size-exclusion chromatography (Supplementary Figures 3A,B). The protein was analyzed by differential scanning fluorimetry (DSF) for its ability to bind adenosine nucleotides (Figure 3A). The melting temperature (Tm) of JAK3 JH2 (apo) was as low as 32.4 ± 0.2°C, indicating inherent lability of the domain but Tm was significantly increased by addition of ATP (ΔTm 8°C with 0.2 mM ATP, 12°C with 1 mM ATP). Supplementing the buffer with either Mg^2+^, Mn^2+^ or Ca^2+^ (in the form of 2 mM of their chloride salts) alone or with ATP did not have a significant effect on the Tm. Adding the monovalent cation salt KCl did not affect the Tm, which was expected as the buffer contained monovalent NaCl. As other JAK JH2s have been shown to require salts to bind ATP, we tested all JAK JH2s with 500 μM ATP with, or without MgCl_2_ (Supplementary Figure 4A) and confirmed, that the Tm of JAK1, JAK2 and TYK2 JH2s increase only if MgCl_2_ is added with ATP, while JAK3 JH2 has higher ΔTm without Mg^2+^ (ATP only). The cation-independency of ATP binding was confirmed by adding 2 mM of the chelating agent EDTA to the buffer, which did not affect the Tm for JAK3 JH2 ATP (Figure 3B).

Measuring the affinity of JAK3 JH2 binding to the fluorescent ATP-analog MANT [2′-(3′)-O-(N-methylanthraniloyl)]-ATP showed that JAK3 JH2 binds MANT-ATP with a dissociation constant (Kd) of 0.4 μM (Figure 3C). Similar values were obtained by titration of the ligand to a constant amount of protein and *vice versa*. IC50 values was determined using fluorescent polarization assay (FP), by competition assay, where ATP competes with ATP pocket binding tracer. The IC50 values for JAK JH2s were compared, and the IC 50 for JAK3 JH2 was 16 μM (without MgCl_2_), indicating tighter ATP-binding affinity for JAK3 JH2 compared to other JAK JH2s (Supplementary Figure 4B, Table 2). Approximation of the JAK3 JH2 Kd was further with DSF, where 50 nM−1 mM ATP was titrated to 3 μM protein. The value was higher than measured with MANT-ATP, namely 40.4 ± 97.1 μM (Supplementary Figure 4C). However, the variation was wide and, as there was no saturation seen when the values were fitted, no Kd was obtained for other JAK JH2s (Supplementary Figure 4C). The differences in the magnitude of the Kd and IC50 values stems from the methods used to evaluate the affinity, e.g., the fact that MANT-ATP uses modified ATP that may affect the binding properties of the molecule. However, both the MANT-ATP and FP methods showed that JAK3 JH2 binds ATP tighter compared to other JAK JH2s, and the binding is cation-independent. Furthermore, the effect of ATP binding to the stability of JAK3 JH2 was greater compared to other JAK JH2s (Supplementary Figure 4C, Table 2).

**Table 2:** Comparison of JAK JH2s.

**JAK JH2**	**Phospho transfer activity**	Kd _(MANT-ATP)^*^_ (References) [μM]	IC50_(FP)^*^_ [μM]	**Tm_Apo_ [^°^C]**	**Tm_ATP_ [^°^C]**	**ΔTm (1 mM ATP) [^°^C]**	**Cations**
JAK2	Weak	1.3 (24)	102	43	46	4.9	Yes
JAK1	No	3.1 (24)	110	46		3.9	Yes
TYK2	No	15 (23)	471	48	48	0.9	Yes
JAK3	No	0.5	16	32	41	9.9	No

* *Kd (MANT-ATP) and IC50 values are shown with MgCl_2_ for JAK1, JAK2 and TYK2 and no MgCl_2_ added for JAK3 JH2*.

Finally, we investigated the binding of ADP and AMP to JAK3 JH2 (Supplementary Figure 4D). ADP showed a significantly reduced ΔTm compared to ATP, and AMP showed no effect on Tm indicating that JAK3 JH2 does not bind AMP and prefers ATP over its de-phosphorylated counterparts. An autophosphorylation assay with radiolabeled ATP did not show incorporation of ^32^P into JAK3 JH2, suggesting that JAK3 JH2 does not possess catalytic activity (Supplementary Figure 4E).

### Mutating the JAK3 JH2 ATP-Binding Site Reverts the Oncogenic JAK3 Hyperactivation

**Figure 4:**
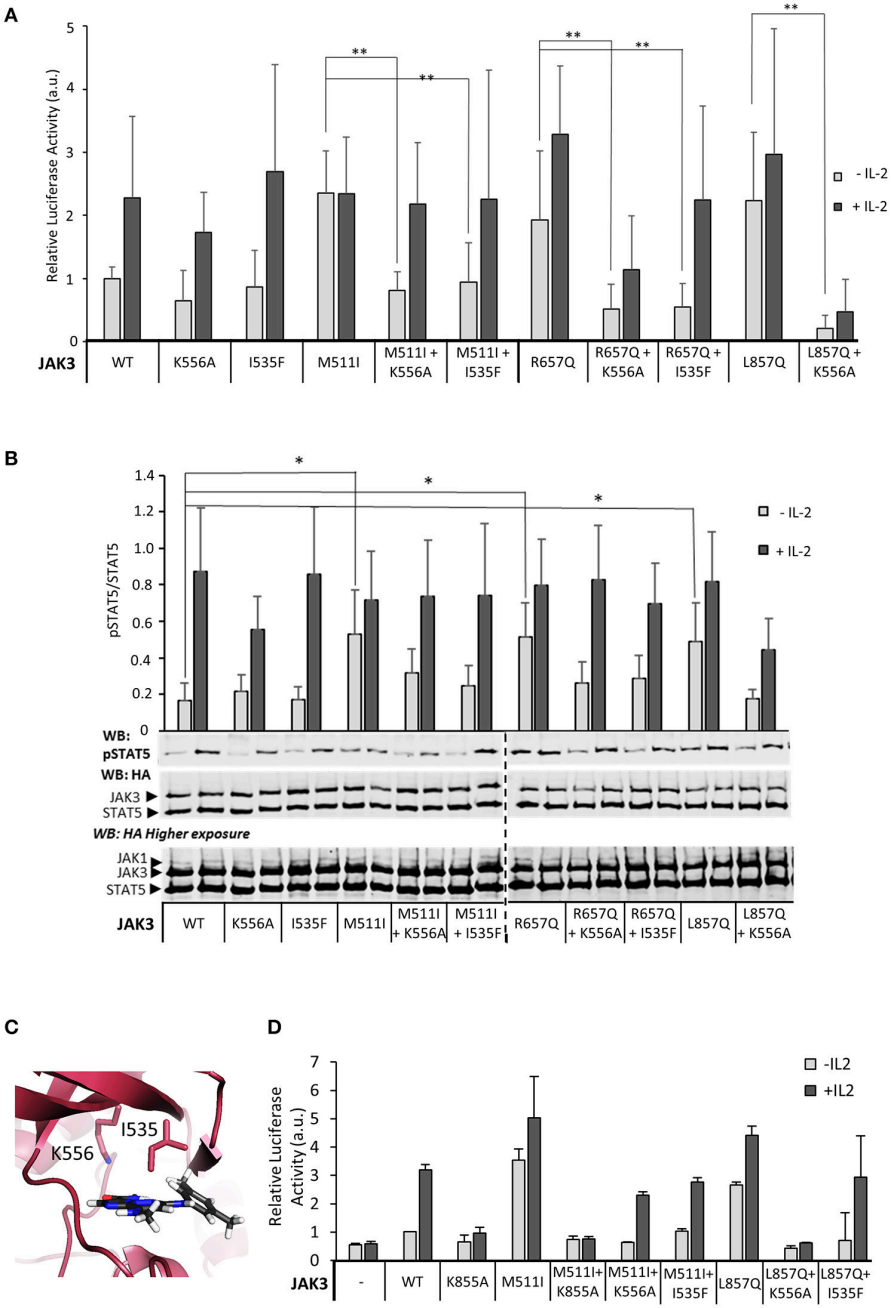
Inhibition of ATP binding to the pseudokinase domain of JAK3 reverts pathogenic, ligand-independent JAK3 signaling, while retaining cytokine-responsiveness. **(A)** JAK3 JH2 ATP-binding site mutations suppress activating JAK3 mutants. Luciferase assay was performed in U4Cγβ cells transfected with JAK1 and the indicated JAK3 mutants using β-galactosidase as a transfection control. The results are from three independent experiment, each having triplicate samples (*n* = 9, expect for K556A *n* = 7). Similar results were obtained with pRL-TK control. Data is normalized to the basal wild-type sample, errors are presented as SD. Statistical analysis of the activating mutations, and their corresponding double mutants were done with two-tailed *t*-test using unequal variances. **p < 0.001. **(B)** Representative Western blot of the transiently transfected U4Cγβ cell lysates showing the pSTAT5 signal. HA-tagged STAT5 was co-transfected with HA-tagged JAK1 and JAK3 and the lysates were blotted with anti-HA and anti-pSTAT5. Lower panel: Quantitative pSTAT5/STAT5 signal analysis. Activation of JAK3 signaling by M511I, R657Q and L857Q is significant (*p < 0.05). Data is average of five experiments. **(C)** The localization of the mutated JAK3 JH2 ATP-binding residues. Homology model of JAK3 JH2 (based on TYK2 structure from PDB, code: 4OLI), ATP is shown in gray. **(D)** Kinase-inactive JAK3 K855A abolishes IL-2 signaling in the presence of the activating JAK3 mutation M511I. Luciferase assay with STAT5-responsive promoter and with pRL-TK control.

Given that JAK3 JH2 has a functional nucleotide-binding site, we wanted to analyze the functional role of ATP binding to JAK3 JH2 in IL-2 signaling. The JAK3 JH2 model identified two residues in the ATP-binding pocket conserved between JAKs (Figure 2A) and suited for mutagenesis. We thus mutated I535 in the β2-sheet of the JH2 N-lobe to a bulkier phenylalanine to sterically inhibit ATP binding. The corresponding JAK2 mutation (JAK2 I559F) has been shown to inhibit ATP binding without affecting the stability of JAK2 JH2 (24). We also mutated the conserved JAK3 JH2 β3 lysine K556, to alanine. The activities of the mutant JAK3 were analyzed in U4Cγβ cells with STAT5-responsive reporter and pSTAT5 analysis. Neither mutation disrupted responsiveness to IL-2 stimulation and the signaling properties resembled wild-type JAK3 (Figure 4).

Inhibition of ATP binding to JH2 by mutations or small molecular weight (smw) inhibitors has been demonstrated to abrogate pathogenic hyperactivation of JAK2 and cytokine signaling via TYK2, respectively (23, 24). To assess the effect of JH2 nucleotide binding in constitutively active JAK3, we constructed double mutants consisting of an activating mutant (M511I, R657Q, or L857Q; see Figure 2) and either of the ATP binding mutants (I535F or K556A). The constructs were transiently transfected and analyzed for STAT5 activation (SPI-luc2 reporter) and pSTAT5 (immunoblotting) (Figures 4A,B,D, Supplementary Figure 5A). In both assays the JH2 ATP-binding site mutations reduced STAT5 activation back to or below wild-type JAK3 levels while retaining cytokine-responsiveness. The sole exception was JAK3 L857Q+K556A, which showed strongly reduced activation by cytokine as well. The more conservative sterically hindering ATP-binding site mutation I535F, however, resulted in wild-type-like STAT5 activation in the L857Q context. IL-2-responsiveness was also regained in M511I+I535F/K556A and R657Q+I535F/K556A (Figures 4A,B). Notably, these results are distinct from complete removal of JAK3 JH2 (ΔJH2, see Figure 1) or removal of JH1 kinase activity by the JAK3 JH1 β3 lysine-to-alanine mutation JAK3 K855A, which resulted in unresponsiveness to cytokine, even when combined with the activating M511I mutation (Figure 4D).

In order to investigate the mechanism of suppression in JAK3 oncogenic signaling by the JH2 ATP-binding mutants, we analyzed phosphorylation of the JAK1 activation loop tyrosine residues. The JH2 ATP binding mutation in JAK3 double mutants reduced JAK1 activation loop phosphorylation compared to activating mutations alone (Supplementary Figure 5C).

The effect of JH2 ATP binding on JAK3 oncogenic mutants was also analyzed using zebrafish (*Danio rerio*). Expression of *rag1*, an IL-2Rγc/JAK3-dependent marker for mature lymphoid cells, was analyzed in JAK3-deficient embryos injected with mRNA encoding JAK3 WT, M511I, I535F or the combined M511I + I535F mutant mRNA as described (36). Each produced a range of *rag1* expression, but both M511I and I535F elicited a small but reproducible enhancement in the mean *rag1* expression compared to WT, which was significantly reduced in the ATP binding deficient M511I + I535F double mutant (Supplementary Figure 6). A similar reduction in *rag1* expression was observed with the double mutant compared to M511I in qPCR experiments performed on embryos from transgenic *lck*::GFP zebrafish injected with JAK3 WT, M511I and M511I + I535F mRNA (data not shown). In contrast, the presence of the I535F mutation did not ablate the induction of *rag1* expression by JAK3 WT in either case (Supplementary Figure 6 and data not shown).

Together, the data indicate that disrupting ATP binding to JAK3 JH2 preserves IL-2-responsiveness but decreases constitutive STAT5 activation, probably by reducing the activating potential of JAK3 to JAK1.

### JAK3 JH2 ATP-Binding Mutations Do Not Inhibit Activation by Pathogenic JAK1 Mutations

We have previously shown that JH2 ATP-binding mutations suppress activating mutations in homomeric cytokine receptor signaling in the same JAK molecule (24). Using the IL-2–JAK1/JAK3 signaling system, we wanted to study the effect of JH2 ATP binding in heterodimeric JAK signaling by assessing whether inhibition of ATP binding in one JAK JH2 could suppress activation by hyperactivating mutations in the other JAK in *trans*. We co-transfected JAK1 JH2 ATP mutant I597F (corresponding to JAK3 I535F) with JAK3 activating mutation M511I, or JAK3 JH2 I535F mutant with the activating JAK1 T-ALL-associated mutation V658F (analogous to the most prevalent MPN-inducing JAK2 mutation V617F) (40). Similar amounts of wild-type and mutated JAK3 and JAK1 proteins were expressed as quantified by western blotting (Supplementary Figure 5B).

**Figure 5:**
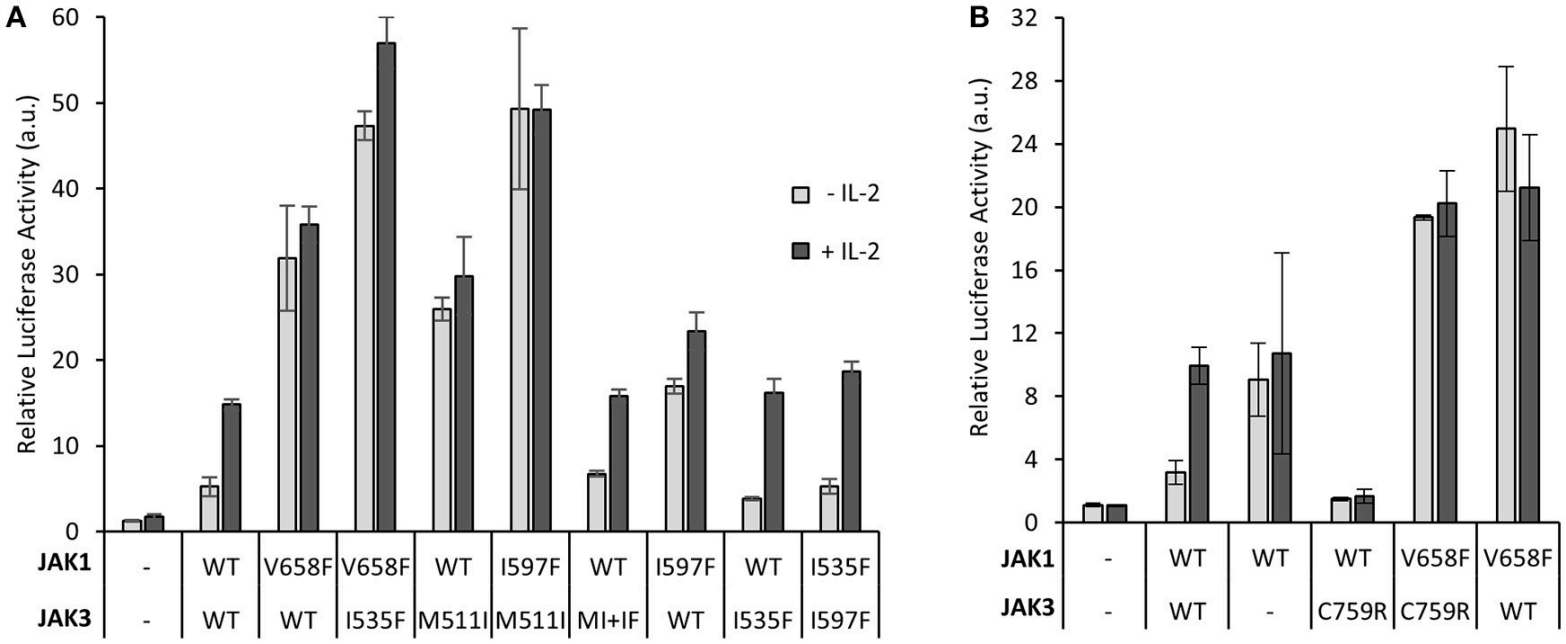
Disrupting the JAK3 JH2 nucleotide binding site inhibits JAK3-induced, but not JAK1-induced, hyperactivation, and *vice versa*. **(A)** Constitutive activation of STAT5 caused by oncogenic JAK3 mutations are suppressed only by ATP-binding site mutations in JAK3 and not its dimerization partner JAK1. Luciferase assay of U4Cγβ cells transiently transfected with JAK1, JAK3, SPI-luc2 and pRL-TK. Results are average from two separate experiments each with triplicate or duplicate (I597F+I535F and I597F+WT JAK3) samples. The results are normalized to the JAK1 WT and JAK3 WT sample. Error bars show the SD. **(B)** Inhibitory JAK3 SCID mutation C759R does not reduce oncogenic hyperaction of JAK1-mutant. Luciferase assay in U4Cγβ cells transiently transfected with JAK1, JAK3, SPI-luc2, and pRL-TK. Error bars are SD of triplicate samples and the data is representative of three independent experiments. Supplementary Figure 5B shows immunoblotting for protein expression levels.

JAK1 JH2 mutant I597F, when co-transfected with JAK3 M511I, did not reduce (hyper)activation and thus did not restore cytokine responsiveness (Figure 5A). In the reverse experimental setting, the JH2 ATP pocket mutation JAK3 I535F could not reduce the activation of constitutively active JAK1 V658F. Together these data suggest that JH2 ATP-site mutations exert their direct suppressing function in *cis* and cannot suppress activation caused by mutations in the other JAK of a heteromeric JAK pair. Mutants disrupting the JH2 ATP-binding possibly function by lowering the kinase activity of JH1 though lowering the stability of the JH2 αC, which interacts with JH1 (24, 32).

To further investigate JAK1–JAK3 interplay on IL-2R, we analyzed the loss-of-function JAK3 SCID mutant C759R (see Figure 2A). The JAK3 C759R mutation completely abrogated signaling of wild-type JAK1 but still failed to inhibit signaling by the hyperactive JAK1 V658F (Figure 5B). Comparable JAK1 and JAK3 expression levels between the mutants and wild-type were confirmed by immunoblotting the samples (Supplementary Figure 5B).

## Discussion

Although JAKs have been intensively studied since their discovery in the late 1980s and early 1990s, many of their molecular regulatory mechanisms are still not fully understood. This notion is particularly true for JAK3, which has nonetheless become a relevant drug target. Importantly, critical information of its regulation is lacking, and for example, structural data exists only for the JAK3 JH1 domain (41). In this study, we focused on characterization of JAK3 JH2 and its function in the context of both normal IL-2R signaling and oncogenic signaling mediated via activating JAK3 mutations. We found that in addition to the well-established functions of the respective JH1s, the JH2s of JAK3 and JAK1 are also required for IL-2 signaling and for hyperactivation of JAK1 and JAK3 oncogenic mutants. Biochemical analysis of recombinant JAK3 JH2 showed that the domain binds ATP but differs from other JAK JH2s in terms of a lower initial Tm, more robust stabilizing effect by ATP, and cation-independency of the nucleotide binding. Furthermore, our cellular signaling analysis suggests that disrupting the ATP-binding site of JAK3 JH2 can inhibit activation caused by JAK3 gain-of-function mutations.

**Figure 6:**
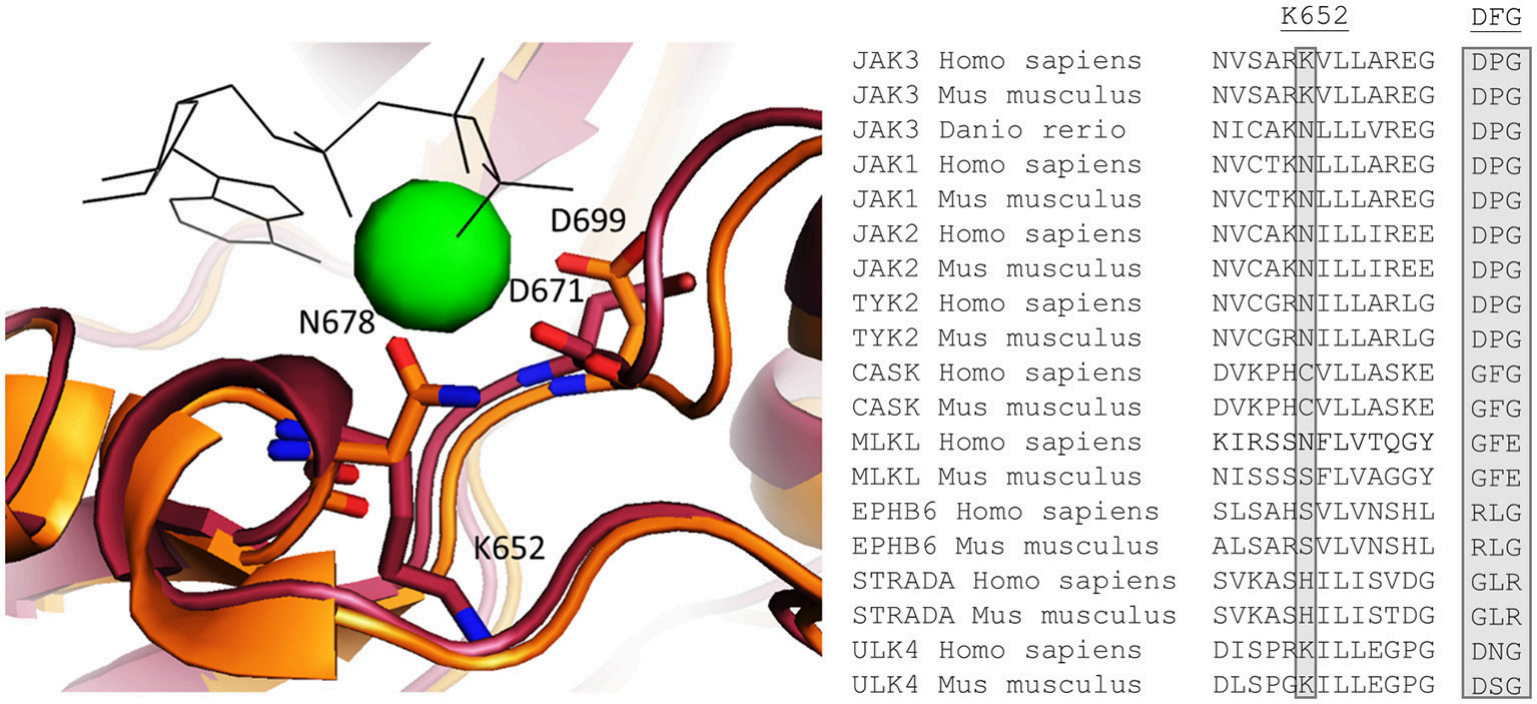
**Left**: Homology model of JAK3 JH2 aligned with the crystal structure of JAK2 [PDB code: 4FVQ (25)]. JAK3 is shown in red and JAK2 in orange. ATP is shown as black shits and the Mg^2+^ present in the JAK2 JH2 structure as a green sphere. **Right**: An alignment of human and mice sequences of JAKs and several other pseudokinases for Mg^2+^ coordination, JAK3 K652, which is conserved as N in other JAKs, and is important for Mg^2+^ coordination, is highlighted. Also the canonical DFG motif are compared and show variation between the pseudokinases.

Our DSF results indicate that JAK3 JH2 binds ATP in a cation-independent manner. In canonical protein kinases such as PKA, Mg-ATP binds in the cleft between the N and C lobes and the two Mg^2+^ ions are coordinated by an aspartic acid in the conserved DFG motif (21). However, pseudokinases are known to possess non-canonical nucleotide binding modes and JAK2 and TYK2 JH2s bind ATP with a single cation coordinated by an upstream asparagine (N678 in JAK2 JH2) (19). Interestingly, this asparagine is conserved also in JAK1, JAK2 and TYK2 JH2s, as well as in the zebrafish (danio rerio) JAK3, but is replaced by a positively charged lysine (K652) in JAK3 JH2 (Figure 6). This difference can explain the cation-independency of ATP binding in JAK3 as it would replace the need for a positively charged cation in coordinating the negatively charged phosphates of ATP. A few other pseudokinases with cation-independent ATP-binding modes have been characterized (19, 21). Of these, MLKL, STRADα, and EphB6 are catalytically inactive, while CASK has been reported to possess phosphotransfer activity (21, 42). Furthermore, ULK4 has been shown to bind nucleotides without cations, but its kinase activity has not been characterized (21). MLKL, STRADα, EphB6 and CASK have divergent sequences in place of the conventional cation-binding DFG motif, namely GFE, GLR, RLG, GFG (all conserved between human and mouse), respectively. In all JAK JH2s, this motif is DPG. In addition, the residue corresponding to JAK3 K652 varies between the above-mentioned proteins: human MLKL has conserved the typical Asn, while ULK4 has a Lys residue similar to JAK3 JH2 and CASK, EphB6 and STRADα all have different residues (Cys, Ser and His, respectively, see Figure 6). These examples illustrate the highly divergent mechanisms for ATP binding that have evolved in pseudokinases (19, 21).

Characterization of recombinant JAK3 JH2 showed an unstable protein with low Tm, but 3-, 6- and 30-fold tighter MANT-ATP binding compared to JAK2, JAK1, and TYK2 JH2s, respectively. A similar trend was also seen in IC50 values derived from a fluorescence polarization competition assay in which a fluorescently-labeled small molecule probe is competed off with unlabeled ATP. In addition, the effect of ATP on the protein stability was considerably larger in JAK3 than in other JAKs (Table 2). Based on the high ATP-binding affinity and lack of kinase activity in JAK3 JH2 (Supplementary Figure 4E), we speculate that ATP binding has a mostly structural role in JAK3 JH2. Furthermore, failure to produce recombinant JAK3 JH2 with mutations designed to disrupt ATP binding (and the success in production of the JAK3 JH2 with the activating R657Q mutation) also indirectly support the notion of a stabilizing effect for ATP (Supplementary Figure 3C).

To assess the effects of ATP binding to JAK3 JH2 on cellular signaling, we constructed mutations into the JH2 ATP binding pocket of full-length JAK3 targeting residues I535 and K556 (see Figures 2, 4). JAK3 JH2 ATP-binding site mutations efficiently suppressed ligand-independent STAT5 activation by JAK3 ALL-mutants R657Q, M511I in JH2, as well as L857Q in JH1 (Figure 4). The suppressing effect of the I535F mutant was further studied in the zebrafish model, where the double mutant (M511I + I535F) induced lower *rag1* expression compared to the M511I mutant (Supplementary Figure 6 and data not shown). In contrast to the kinase-inactive form of JAK3 (K855A), which lowered both ligand-independent basal activity and IL-2 induced signaling, the JH2 ATP-binding site mutations only suppressed ligand-independent STAT5 activation, while retaining IL-2-dependent STAT5 activation. The only exception to this was the double mutant L857Q + K556A, which showed lowered signaling capacity for as of yet unknown reasons.

Based on homology modeling the JAK3 JH2 gain-of-function mutations R657Q and M511I reside in the autoinhibitory JH1–JH2 interface (see Figure 2 for suggested JH2–JH1 interaction pair between JAK3 R657 and D865), which, when disrupted, is likely to cause increased JAK3 JH1 basal tyrosine kinase activity (27, 32). This, in turn activates JAK1, which finally acts as the effector kinase phosphorylating STAT5 (3, 43) and leading to ligand-independent activation of the IL-2-STAT5 pathway. Our results presented here corroborate this hypothesis as we show that functionally sound JAK1 (including both JH1 and JH2) is required for STAT5 activation also by clinical gain-of-function JAK3 mutations (3, 43).

In contrast to the JH2 mutations, the L857Q, which is located next to the catalytically important K855 in JH1 (see Figure 2) and thus unlikely to disrupt the JH1–JH2 interaction, might activate JH1 more directly by e.g., repositioning the JH1 C-helix or the nearby DFG motif into a more active conformation. Our data shows that L857Q is still dependent on JAK1 JH1/JH2, thus the mutation does not seem to result in sufficiently high JAK3 JH1 kinase activity to lead to JAK1-independent phosphorylation of STAT5 as has been suggested for a proline-mutation of the same residue: L857P (43). Interestingly, our results also show that L857Q is suppressed by JAK3 JH2 ATP-binding site mutations, thus showing clear commonalities in activation mechanisms of JAK3 JH2 and JH1-borne gain-of-function mutations. Molecular dynamics simulations of JAK2 JH2 have suggested that ATP binding stabilizes the N lobe of C helix (αC) in JH2 (24), and this is likely to be the case for JAK3 as well. Whether this stabilization causes strengthening of the JH2-JH1 interaction (and thus suppression of gain-of-function mutations), or whether ATP binding to JH2 stabilizes a conformation of JH2 that enables a distinct activating interaction involved in ligand-independent activation of JAKs, is currently not unequivocally solved (24).

In addition to activating JAK3 mutations, we studied the SCID-related loss-of-function mutation JAK3 C759R. JAK3 C759R has been shown to be deficient in phosphorylating STAT5 and STAT3 and lacks kinase activity, but to be constitutively phosphorylated on the JH1 activation loop (JAK3 Y980-981) and bind to its receptor IL-2Rγ (31). JAK3 JH2 homology modeling suggests that mutation of C759 on α-helix H (see Figure 2C) to the bulkier and cationic Arg is likely to cause severe disruption of the JH2 C-lobe. Indeed, JAK3 C759R showed strongly reduced STAT5 activation and the effect was comparable to JAK3 ΔJH2 or kinase-dead JAK3 K855A.

Currently it is not clear, whether inhibition of JH1 by JH2 occurs in *cis* or *trans* in physiological JAK dimers, and data supporting both hypotheses has been presented (9). However, the interplay between JAK1 and JAK3 is evident in hematological malignancies, and cells transformed by activating JAK1 mutations become resistant to JAK inhibitors by acquiring activating mutations in JAK3 and *vice versa* (44). Considering the tight JAK1–JAK3 interplay on IL-2R, we studied the possible cross-regulation between JAK1 and JAK3 JH2s and observed that mutating the JAK1 JH2 ATP binding site could not inhibit activation caused by JAK3 M511I, which was also the case in the reversed experimental setting (JAK3 JH2 ATP-site mutant transfected with constitutively active JAK1 V658F). These data suggest that any suppressing effect caused by disrupting the JAK3 JH2 ATP-binding site is exerted only in *cis* and not between JAK3 and JAK1. However, as noted before, JAK1 is the dominant effector in STAT5 activation, and thus the overexpression of JAK1 in our experimental setup may compensate for any inhibitory effects of a JAK3 JH2 ATP binding mutant. Furthermore, in our experimental approaches we have used rational mutagenesis and deletion/recombinant constructs, and although the constructs have been designed based on best available information they are not natural proteins and thus our experimental systems bear inherent limitations.

We also tested the JAK1–JAK3 interaction by co-transfecting the inhibitory JAK3 SCID mutant C759R with JAK1 V658F. Again, JAK1 dominancy was apparent and JAK3 C759R could not reduce the activation of constitutively active JAK1 V658F even though it did inhibit IL-2-induced activation of wild-type JAK1. Together, these data support a model in which JAK3 acts as a regulating initiator kinase mainly responsible for activation of JAK1, which subsequently phosphorylates downstream targets. If JAK1 is activated by other means, like constitutively activating JAK1 mutations, any activating functions of JAK3 become unnecessary. Notably, the reverse is not true and even mutationally activated JAK3 relies on JAK1 for effector functions.

The vast majority of JAK inhibitors currently in use or development target the kinase domain of JAKs in its active, DFG-in, conformation, and they often suffer from poor selectivity (45). Novel JAK3 inhibitors have been developed that bind to a JAK3-specific, non-catalytic cysteine (C909) in the JH1 domain, thus enabling significant selectivity toward JAK3 (41, 46). Recently, an ATP binding competitive inhibitor against JH2 of TYK2 was shown to have high specificity and efficacy in IL-23 and type I IFN signaling (47). Our results suggest that the JAK3 JH2 ATP pocket may also be a relevant therapeutic target in diseases caused by JAK3 hyperactivation.

## Ethics Statement

All experiments performed were approved by the Deakin University Animal Welfare Committee.

## Author Contributions

JR, AV, and VB planned and performed the experiments (AV contributed to the FP measurement and VB performed the *in vivo* experiments). HH devised and planned the experiments. JR and HH analyzed the data and drafted the manuscript. OS and AW supervised the work and edited the manuscript.

### Conflict of Interest Statement

The authors declare that the research was conducted in the absence of any commercial or financial relationships that could be construed as a potential conflict of interest.
