# Supplementary material for: Hyperactivation of Oncogenic JAK3 Mutants Depend on ATP Binding to the Pseudokinase Domain
Source: Front Oncol. 2018 Dec 3;8:560. doi: 10.3389/fonc.2018.00560 (PMC6287396; doi:10.3389/fonc.2018.00560)

Supplementary Material

**Hyperactivation of oncogenic JAK3 mutants depend on ATP binding to the pseudokinase domain**

Juuli Raivola^1^, Henrik M. Hammarén^1^, Anniina T. Virtanen^1^, Vilasha Bulleeraz^4^, Alister C. Ward^4^, Olli Silvennoinen^1-3*^

^1^Faculty of Medicine and Life Sciences, University of Tampere, Arvo Ylpön katu 34, FI-33014 Tampere, Finland.

^2^Fimlab Laboratories, Pirkanmaa Hospital District, FI-33520 Tampere, Finland.

^3^ Institute of Biotechnology, University of Helsinki, P.O.Box 56 (Viikinkaari 5), FI-00014, Helsinki, Finland.

^4^ School of Medicine, Deakin University, Geelong, Victoria 3216, Australia; Centre for Molecular and Medical Research, Deakin University, Geelong, Victoria 3216, Australia.

*** Correspondence:**Corresponding Author
olli.silvennoinen@uta.fi

## Supplementary Figures

**Figure S 1: JAK1 mediates cytokine-independent activation of STAT5, but both JAK1 and JAK3 are required for IL-2-dependent STAT5 activation**

1. STAT5 activity when varying amounts of JAK1 and JAK3 were transfected in U4Cγβ cells. STAT5 responsive promotor SPI-luc2 and pRL-TK control plasmid were also included. Values under the bars show the amount of plasmid transfected in ng per 96-well plate well.
2.
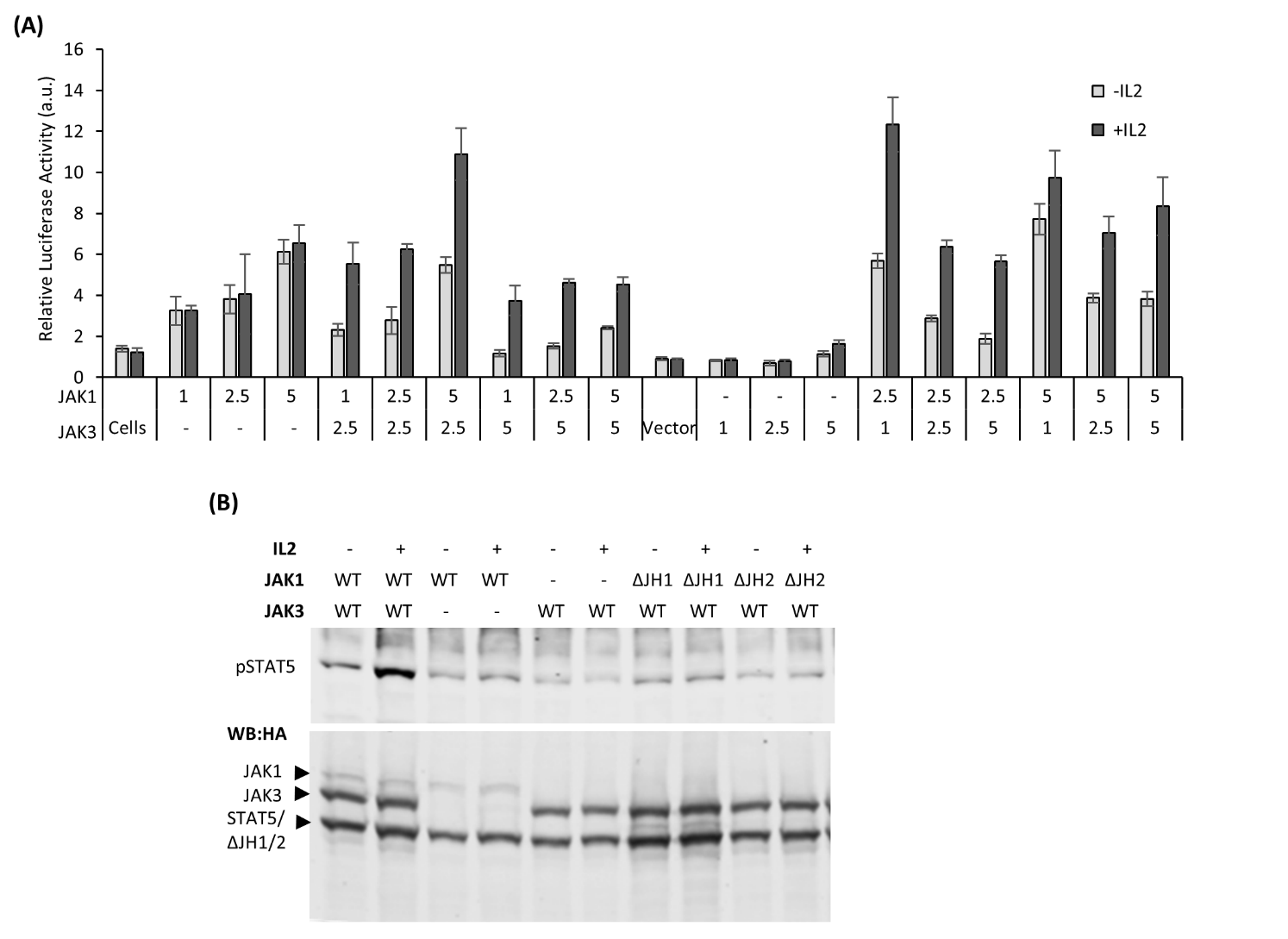
Immunoblots of U4Cγβ cells transfected with wild-type HA-tagged JAK3 and/or JAK1 and STAT5. Blots were labelled with HA and pSTAT5 antibodies. The ratio of the pSTAT5/STAT5 (HA) signal is shown as a bar chart.

**Figure S 2: Expression levels of the JAK1 and JAK3 mutants**

1. Constitutively active JAK3 mutants cannot signal without JAK1. STAT5-responsive promoter with activating JAK3 mutants without JAK1 were transfected into U4Cγβ cells.
2. Western blot of the JAK1 domain deletions with JAK3 activating mutants. Cells were transiently transfected with different HA-tagged JAK1, JAK3 and STAT5 constructs, and detected with HA and pSTAT5 antibodies.
3.
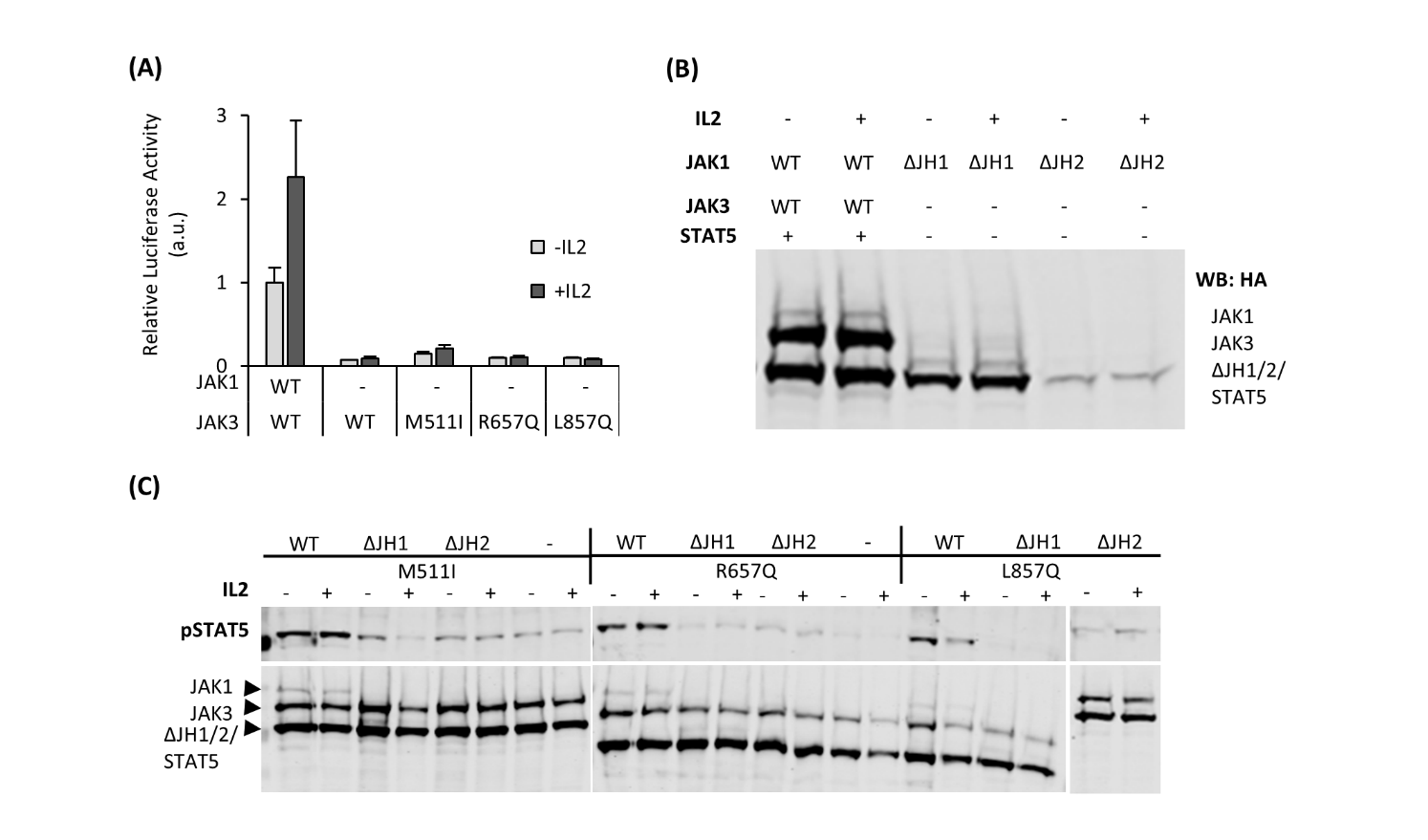
Expression analysis of JAK1 domain deletions. JAK1ΔJH1 and ΔJH2 are same size with STAT5 and to see the expression of the constructs, the cells were transfected with the JAK1 constructs with and without STAT5 and labelled with HA antibody. The expression of full-length JAK1, JAK3 and STAT5 are shown as a control.

**Figure S 3: Purification and kinase activity of the recombinant JAK3 JH2**

1. Purification of the recombinant JAK3 JH2 (511-790 C-terminal His). Elution (UV-signal) profile of the SEC purification. Peak 2 includes the fraction with JAK3 JH2 and peak 1 the void.
2. Fractions corresponding to the peak 2 in the UV detector were collected and run to 12% SDS-PAGE gel. The size of the band in the gel corresponds to JAK3 JH2 (33 kDa). Also shown SDS-PAGE from the Ni-NTA affinity purification
3. Ni-NTA purification of the Mutant JAK3 JH2 constructs. From right to left, wild type JAK3 JH2, ATP-binding site mutants I535F and K556A and activating mutant R657Q. Three elutions from the beads are shown. Gels of I535F and R657Q include also un-bound (UB) sample. Right: List of JAK3 JH2 mutants designed to inhibit ATP-binding. No protein was obtained from the mutants.


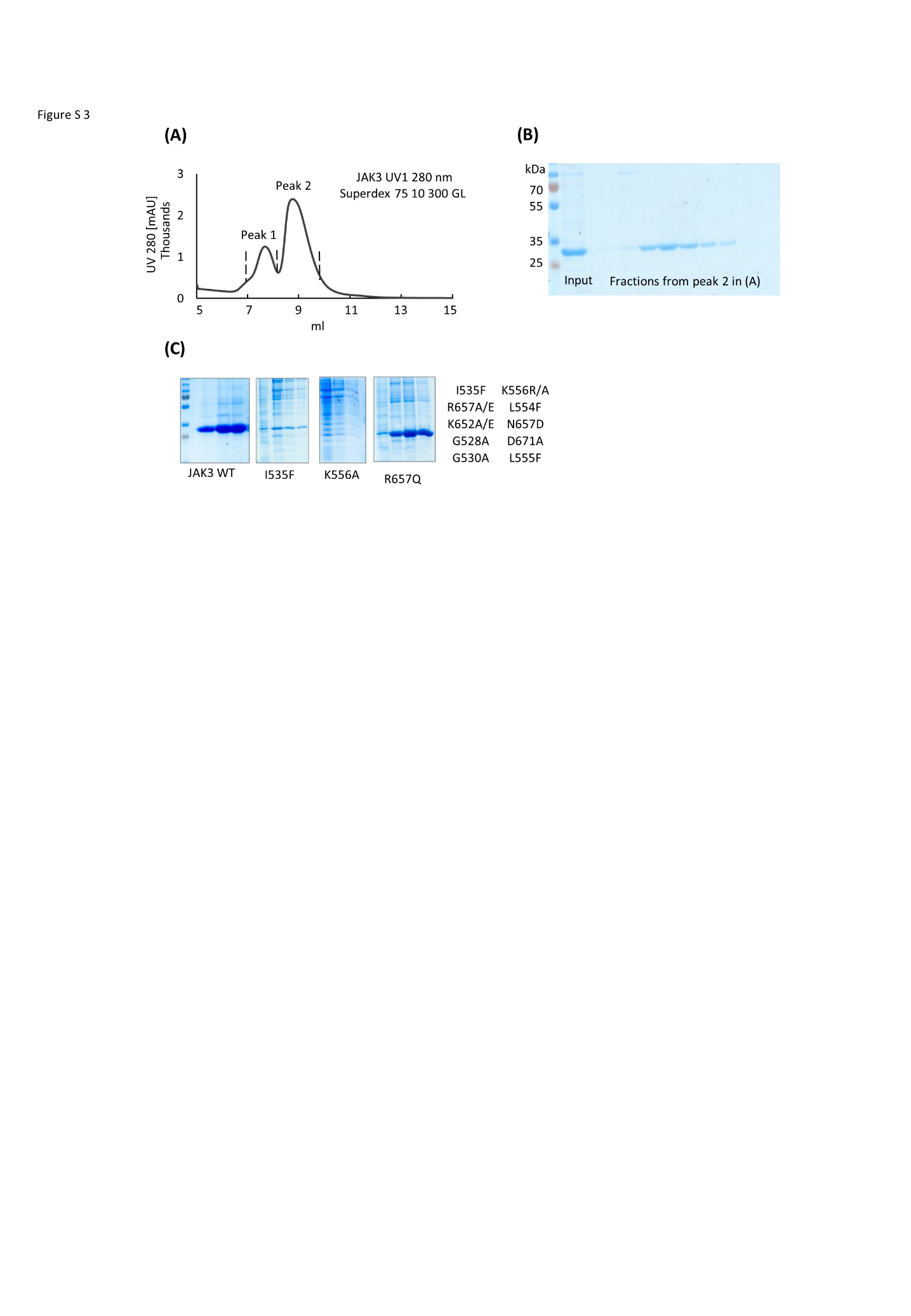


**Figure S 4: Analysing the ATP binding of recombinant JAK3 JH2**

1. Comparison of the cation dependency of JAK JH2s with DSF. Melting temperature of different JAK JH2s was measured with or without ATP and MgCl2. 3 µM protein was used with 500 µM ATP and the ΔTm was compared to ATP + 1 mM MgCl2. Errors are SD from triplicate samples.
2. IC50 values of JAK JH2 ATP binding. ATP was competed with fluorescently labelled tracer, which binds to the JH2 ATP binding pocket and the binding curves for all JAK JH2s were determined by ATP titration, with and without MgCl2. All proteins were run in triplicates and the data with SD errors are shown.
3. Left panel: ATP titrationin DSF with all JAK JH2s. Effect of ATP to the melting temperature is presented as ΔTm values. Errors are SD from triplicate samples. Right panel: Kd value for JAK3 JH2 was determined from the ATP titration curve shown in the left panel.
4. JAK3 JH2 binds preferentially ATP. Nucleotide titration with ATP, ADP and AMP (0.2, 0.5, 1 mM concentrations) and the stability of the protein was analyzed with DSF. All bar graphs in (A), (B) and (C) show average ΔTm from duplicate samples. Error bars represent SD.
5. Autophosphorylation assay of recombinant JAK3 JH2. 0.05 µM of radioactively labelled γ^32^P-ATP (10 µCi/µl) was incubated 20, 40, 80 or 180 min with 0.6 µM JAK3 JH2 or with a positive control (0.3 µM of active tyrosine kinase, EGFR, supplemented with 20 mM MgCl2). A sample without γ^32^P-ATP was used as a negative control. The faint bands in JAK3 JH2 + ATP (indicated as arrowheads) are the same size as in the EGFR and thus considered to be caused by impurities (other kinase) in the sample. No phosphotransferase activity was detected in the negative control.


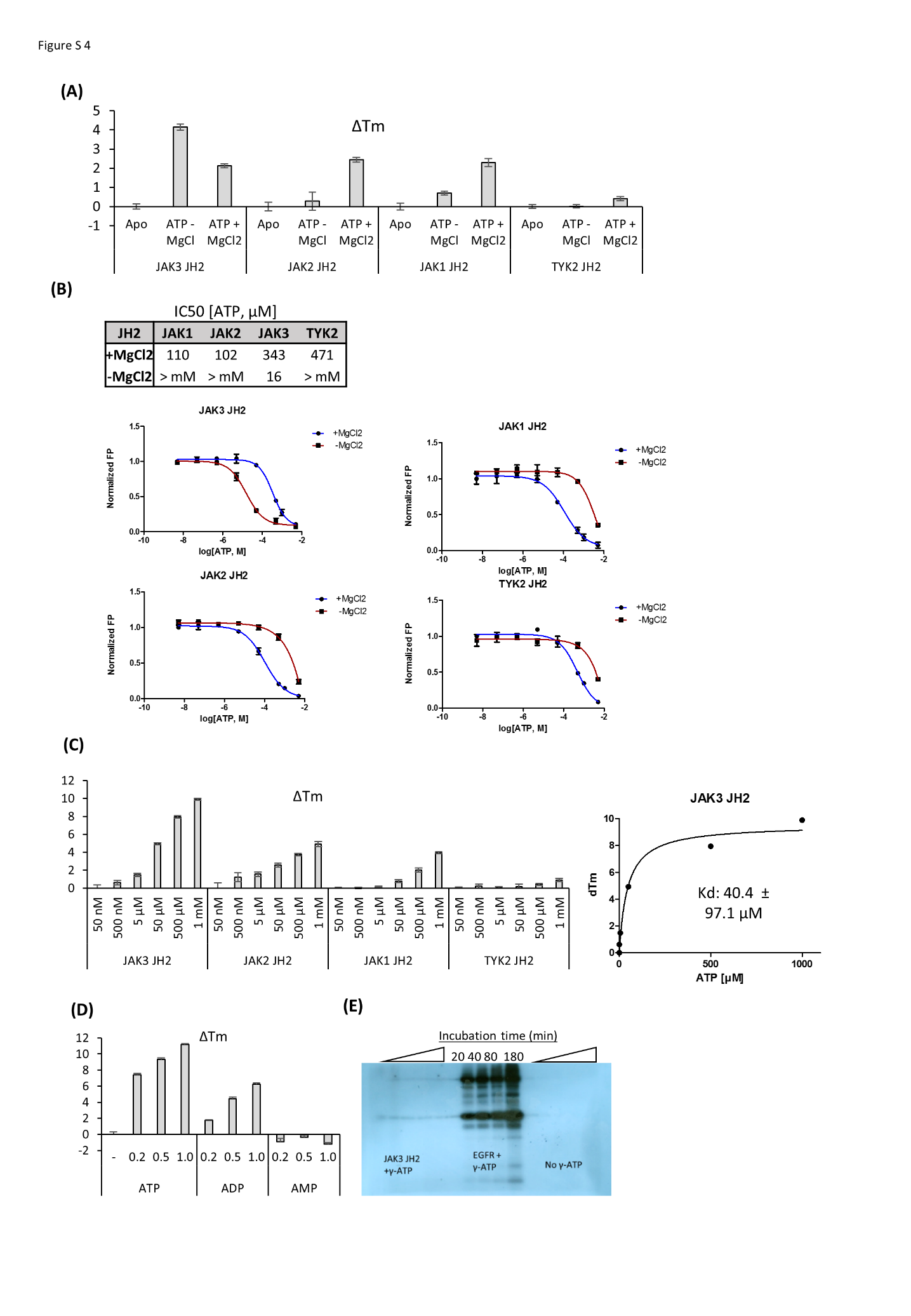


**Figure S 5: Whole blots from Figure 4 and 5 with pJAK1 analysis**

1. Representative whole blots form the pSTAT5 analysis of the JAK3 ATP binding, activating, and double mutants shown in Figure 4.
2. Analysis of JAK1 phosphorylation. pJAK1 antibody was used to detect pJAK1 status with different JAK3 mutants. Lower panel: Quantitative analysis from the blots done by dividing the pJAK1 signal with HA-signal from HA-tagged JAK1.
3. Representative blots from experiments used in Figure 5. HA-tagged JAK1 and JAK3 expression levels were detected with HA-antibody. Dotted lines between blots indicate separate gels.


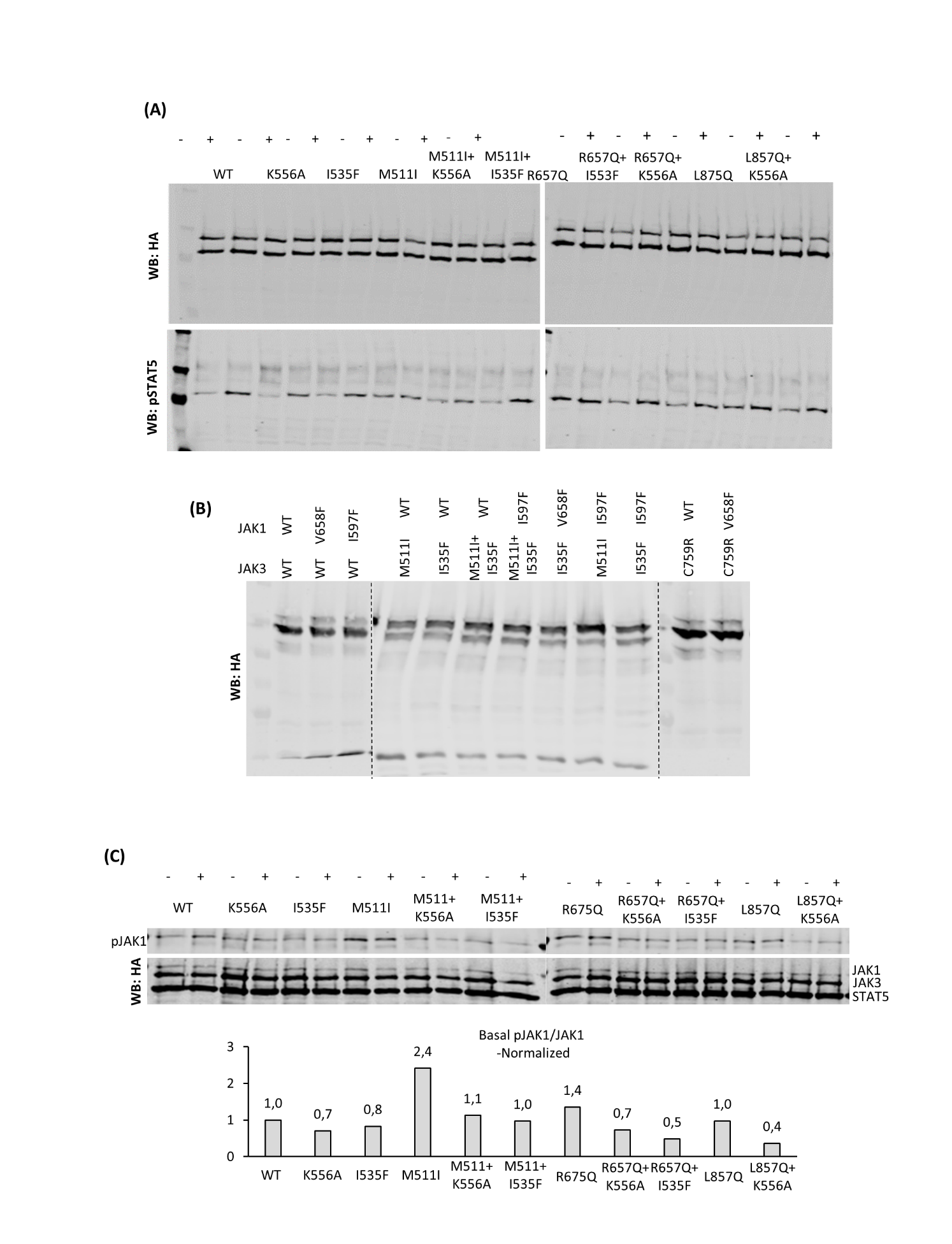


**Figure S 6: Mutation of JAK3 JH2 ATP site ablates the effects of activating mutation in vivo.**

Relative expression of the IL-2Rγc/JAK3-dependent lymphoid marker *rag1* at 5 dpf in *jak3^-/-^* mutant embryos injected with mRNA encoding zebrafish JAK3 wild-type (WT) (n=29) and mutants M511I (n=26), I535F (n=21) and M511I+I535F (n=6), as indicated. Data is presented as mean ± SEM, with statistical significance indicated (* *p* < 0.05).


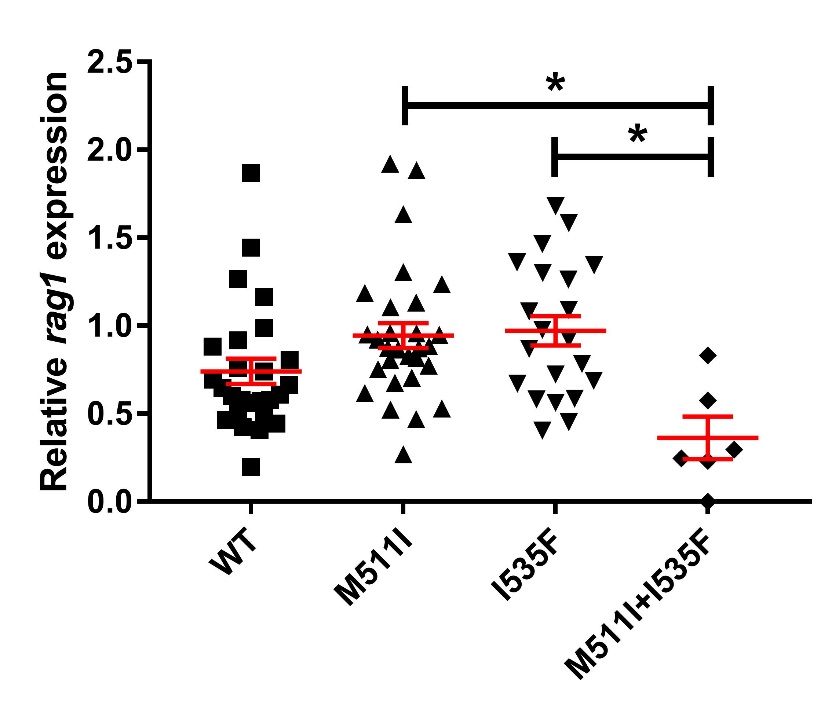

Supplement: Supplementary file 1 [file Data_Sheet_1.docx]
